# KRAS as a Modulator of the Inflammatory Tumor Microenvironment: Therapeutic Implications

**DOI:** 10.3390/cells11030398

**Published:** 2022-01-24

**Authors:** Flávia Pereira, Anabela Ferreira, Celso Albuquerque Reis, Maria João Sousa, Maria José Oliveira, Ana Preto

**Affiliations:** 1 Centre of Molecular and Environmental Biology (CBMA), Department of Biology, Campus de Gualtar, University of Minho, 4710-057 Braga, Portugal; flaviabrandao.fcbp@gmail.com (F.P.); aaferreira18@gmail.com (A.F.); mjsousa@bio.uminho.pt (M.J.S.); 2 Institute of Science and Innovation for Bio-Sustainability (IB-S), Campus de Gualtar, University of Minho, 4710-057 Braga, Portugal; 3 Institute for Research and Innovation in Health (i3S), University of Porto, 4200-135 Porto, Portugal; celsor@ipatimup.pt (C.A.R.); mariajo@ineb.up.pt (M.J.O.); 4 Institute of Biomedical Engineering (INEB), University of Porto, 4200-135 Porto, Portugal; 5 Institute of Biomedical Sciences Abel Salazar (ICBAS), University of Porto, 4050-313 Porto, Portugal; 6 Institute of Molecular Pathology and Immunology, University of Porto (IPATIMUP), 4200-135 Porto, Portugal

**Keywords:** *KRAS* mutations, tumor microenvironment, inflammation, pancreatic cancer, colorectal cancer, lung cancer, therapy

## Abstract

*KRAS* mutations are one of the most frequent oncogenic mutations of all human cancers, being more prevalent in pancreatic, colorectal, and lung cancers. Intensive efforts have been encouraged in order to understand the effect of *KRAS* mutations, not only on tumor cells but also on the dynamic network composed by the tumor microenvironment (TME). The relevance of the TME in cancer biology has been increasing due to its impact on the modulation of cancer cell activities, which can dictate the success of tumor progression. Here, we aimed to clarify the pro- and anti-inflammatory role of *KRAS* mutations over the TME, detailing the context and the signaling pathways involved. In this review, we expect to open new avenues for investigating the potential of *KRAS* mutations on inflammatory TME modulation, opening a different vision of therapeutic combined approaches to overcome KRAS-associated therapy inefficacy and resistance in cancer.

## 1. Introduction

**Figure 1:**
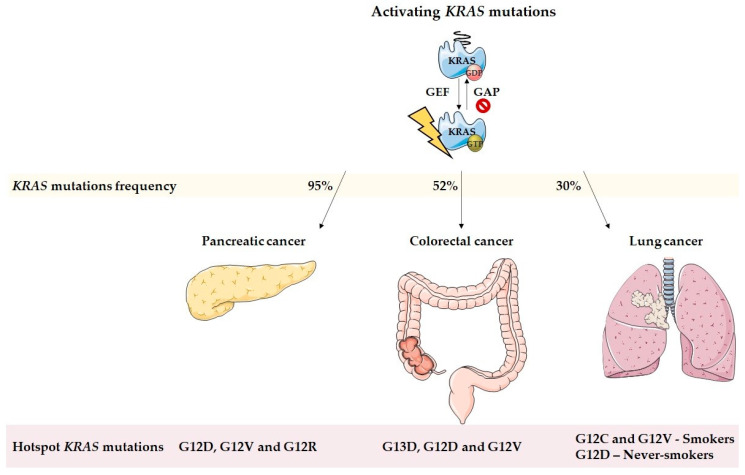
*KRAS* mutations in pancreatic, colorectal, and lung cancer. *KRAS* mutations are one of the earliest major events in pancreatic cancer, found in over 95% of cases. Among *KRAS* mutations, G12D and G12V are the most frequent alterations, followed by G12R. In colorectal cancer, *KRAS* mutations are present in about 52% of cases. Oncogene *KRAS* activating mutations G13D, G12D, and G12V are the most frequent in this type of cancer. In lung cancer, activating *KRAS* mutations are found in over 30% of cases and are one of the most prevalent mutations associated with tobacco exposure. Among those, G12C and G12V mutations are the most associated with patients who smoke, whereas G12D is mainly found in never-smokers.

Kristen rat sarcoma viral oncogene homolog (*KRAS*) belongs to the human Ras genes family, which also comprises the Harvey rat sarcoma viral oncogene homolog (*HRAS*) and the Neuroblastoma rat sarcoma viral oncogene homolog *(NRAS*). These genes encode 4 highly related proteins with 90% similarity, namely, KRAS4A, KRAS4B, HRAS, and NRAS [1,2,3,4]. KRAS4A and KRAS4B are two splice variants, KRAS4B being the dominant form in human cells and here, referred to as KRAS [4]. KRAS is a small GTPase that functions as a signal transducer from extracellular stimuli-activated cell surface receptors to diverse well-regulated cytoplasmic signaling networks, such as the mitogen-activated protein kinase (MAPK) and the phosphoinositide 3-kinase (PI3K) [1,5]. Wild-type KRAS proteins are GDP and GTP binary proteins with on-off switches well regulated by guanine-nucleotide exchange factors (GEFs) and by GTPase activating proteins (GAPs). Whereas GEFs stimulate the formation of active Ras-GTP, GAPs accelerate the hydrolytic GTPase activity, promoting the release of GTP from RAS-GTP and the formation of the inactive RAS-GDP [1,2,3,5,6,7]. However, when KRAS proteins are mutated, they become insensitive to GAPs inactivation and remain in the active GTP-bound state, leading to the constitutive activation of downstream Ras signaling pathways [5,8,9,10]. Therefore, these constitutively active KRAS proteins contribute to self-sufficiency in growth signals, increase of cell proliferation, suppression of apoptosis, increase in autophagy, altered cell metabolism, and changes in the tumor microenvironment (TME) [1,5,10,11,12,13,14]. Interestingly, mutations in the Ras family are one of the most common oncogenic events (33%), *KRAS* mutations being the most frequent (21.6%), followed by *NRAS* (8.0%) and *HRAS* (3.3%) [3]. Amongst all human cancers, pancreatic, colorectal, and lung are the ones with a higher percentage of *KRAS* mutations (Figure 1) and constitute, therefore, the focus of our review [1,3,9].

In 2020, pancreatic cancer was the seventh worldwide leading cause of cancer-related deaths in both sexes and accounted for approximately as many cases (496,000) as deaths (466,000) [15]. Pancreatic cancer is a highly aggressive malignancy associated with a particularly poor prognosis that exhibits a median survival of fewer than 6 months and a 5-year survival rate of 3 to 5% [16,17]. *KRAS* mutations are one of the earliest and most serious events in pancreatic cancer and are found in over 95% of the cases [5,16,18,19]. Among *KRAS* mutations, the *KRAS*^G12D^ (39.2%) and the *KRAS*^G12V^ (32.5%) are the most frequent alterations, followed by the *KRAS*^G12R^ (17.1%) [20,21,22]. 

Colorectal cancer was, in 2020, the third most frequently diagnosed cancer and the second leading cause of cancer-related deaths in both sexes worldwide [15]. *KRAS* mutations are present in about 52% of colorectal cancer cases and are in the top 5 of mutated genes in 2 different databases, namely, the International Cancer Genome Consortium (ICGC) and The Cancer Genome Atlas (TCGA), along with *APC*, *TP53*, and *TIN* [1,23,24,25,26]. Oncogene *KRAS* activating mutations *KRAS*^G13D^, *KRAS*^G12D^, and *KRAS*^G12V^ are the most frequent mutations in colorectal cancer, with the codon 12 being the most affected [8,12,25,27,28,29]. *KRAS* mutations can also be caused by substitutions in codons 13, 61, 117, and 146. These mutations are an early event in colon carcinogenesis and are well conserved between primary tumor and corresponding metastases [14,30,31]. *KRAS* mutations have also been associated with poor overall survival and increased tumor aggressiveness [25].

In 2020, lung cancer was the second most commonly diagnosed cancer worldwide and the leading cause of cancer-related deaths [15]. Activating *KRAS* mutations are found in over 30% of lung cancer cases and are one of the most prevalent mutations associated with tobacco exposure [32,33,34,35,36,37]. Among those, *KRAS*^G12C^ and *KRAS*^G12V^ are the most associated with patients who smoke, whereas *KRAS*^G12D^ is mainly found in patients who had never smoked [9,10,20,38]. In addition, *KRAS* mutations are present in more than 35% of cases of the non-small-cell lung cancer subtype, which is the most frequent form of lung cancer (85%) [32,36]. 

Epidemiological and clinical studies have shown a strong relationship between lung cancer, inflammatory microenvironment, and chronic infection [39], as well as between colorectal cancer and chronic inflammatory diseases [24]. In pancreatic cancer, an extensive stromal remodeling, with inflammatory cells and fibrotic scars, is also a hallmark of this type of cancer. *KRAS* mutations have been tightly associated with modulation of tumor inflammation, which has been gradually recognized as a key contributor for tumorigenesis by affecting the immune response, as well as the efficacy of treatments [1,40]. Therefore, exploring how cancer cells harboring oncogenic *KRAS* mutations may instigate the inflammatory TME, leading to chronic inflammation and stroma remodeling, is of extreme relevance [16,24,41].

This review summarizes the intensive efforts made to understand the effects of *KRAS* mutations, not only on cancer cells, but also on the TME, detailing the context and the signaling pathways involved. Additionally, by exploring the impact of *KRAS* mutations on the inflammatory TME, we expect to open new avenues for investigating the potential of these mutations on the TME modulation, opening a new vision of combined therapeutic approaches to overcome KRAS-associated therapy inefficacy and/or resistance in cancer.

## 2. KRAS and the Inflammatory Tumor Microenvironment Modulation

**Figure 2:**
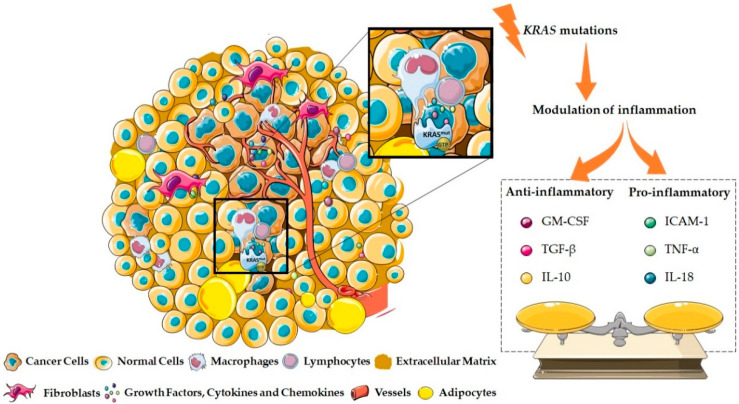
KRAS as a crucial TME modulator. The TME is composed, not only of tumor cells, but also several non-tumor stromal elements such as immune cells, fibroblasts, adipocytes, endothelial cells, neurons, osteoblasts, osteoclasts, and ECM components. This dynamic, challenging microenvironment modulates and can be modulated by several factors, namely, *KRAS* mutations. Several studies have reported that *KRAS* mutations can drive the secretion of anti-inflammatory cytokines, such as IL-10, TGF-β, and GM-CSF, with the ability to sustain an immunosuppressive TME and to promote tumor progression. Other studies have also demonstrated that *KRAS* mutations may interfere with the secretion of pro-inflammatory cytokines, with an anti-tumor effect, such as ICAM-1, TNF-α, and IL-18. Thus, KRAS seems to act as a modulator of both an anti-inflammatory and a pro-inflammatory TME.

The TME is a dynamic network composed, not only by tumor cells, but also by several non-tumor cell types, including stromal cells as immune cells (macrophages, neutrophils, dendritic and natural killer cells, myeloid-derived suppressor cells (MDSCs), B and T cells), fibroblasts, adipocytes, endothelial cells, neurons, osteoblasts, osteoclasts, and the extracellular matrix (ECM). This non-cellular component, together with the tumor and the non-tumor cells, establish a dynamic, challenging microenvironment that can be modulated, but especially modulates cancer cell activities, dictating the success of tumor progression (Figure 2) [1,42,43,44].

Inflammation has been gradually recognized as a key initiator and contributor for tumorigenesis by orchestrating the immune surveillance and the immune escape, but also by affecting treatment response [1,40]. Interestingly, the concept of tumor-promoting inflammation has been tightly associated with *KRAS* mutations [1]. In fact, in colorectal cancers, the majority of the cases with a high prevalence of *KRAS* mutations correlate with chronic inflammatory diseases [24]. KRAS and its downstream interactors are described as capable of shaping the immune microenvironment through the induction of the nuclear factor kappa light chain enhancer of activated B cells (NF)-kB signaling, which in turn promotes the transcription of several cytokines and chemokines, including interleukin (IL)-1α/β, IL-6, tumor necrosis factor α (TNF-α), Cys-X-Cys Chemokine (CXCL)-1, 2, 5, and 8, monocyte chemoattractant protein 1 (MCP-1 or CCL2), inducible nitric oxide synthase (iNOS), intracellular adhesion molecule 1 (ICAM-1), and endothelial leukocyte adhesion molecule 1 (ELAM1) [1,32]. Independently of NF-kB, KRAS-downstream partners, such as RAF/MAPK and PI3K, may also induce IL-10, transforming growth factor β (TGF-β) and granulocyte-macrophage colony-stimulating factor (GM-CSF) expression [1,32]. Several studies already reported that *KRAS* mutations could drive the secretion of anti-inflammatory cytokines, such as IL-10 and TGF-β, with the ability to sustain an immunosuppressive TME, whereas other studies verified that *KRAS* mutations could interfere with the secretion of pro-inflammatory cytokines, such as ICAM-1, TNF-α, IL-1β, IL-6, and IL-18 (Figure 2) [18,32,45,46]. Thus, KRAS seems to act as a modulator of both an anti-inflammatory and a pro-inflammatory TME. In this section, the ability of *KRAS* mutations to modulate the acquisition of a pro- as well as an anti-inflammatory TME is discussed in detail.

### 2.1. KRAS as a Pro-Inflammatory Modulator of the Tumor Microenvironment

**Figure 3:**
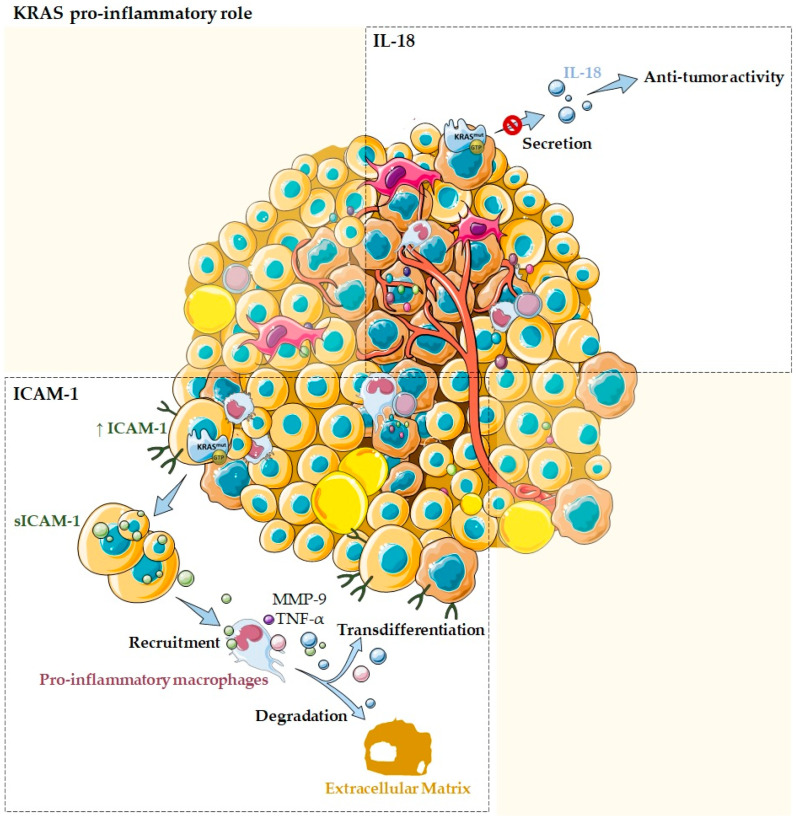
KRAS as a pro-inflammatory tumor microenvironment modulator. Several studies reported the association of KRAS with pro-inflammatory cytokines and chemokines, such as ICAM-1 and IL-18. Normal acinar cells transfected with oncogenic mutant *KRAS* are described to express high levels of ICAM-1, which is then secreted into its soluble form. The sICAM-1 acts as a chemoattractant for pro-inflammatory macrophages and stimulates them to produce MMP-9 that allow ECM degradation, as well as pro-inflammatory chemokines, such as TNF-α that can drive transdifferentiation signaling. *KRAS* mutations can also modulate the TME by impairing IL-18 secretion, blocking its immune-stimulatory function and, thus, contributing to evasion of the local immune system during tumor development.

**Figure 4:**
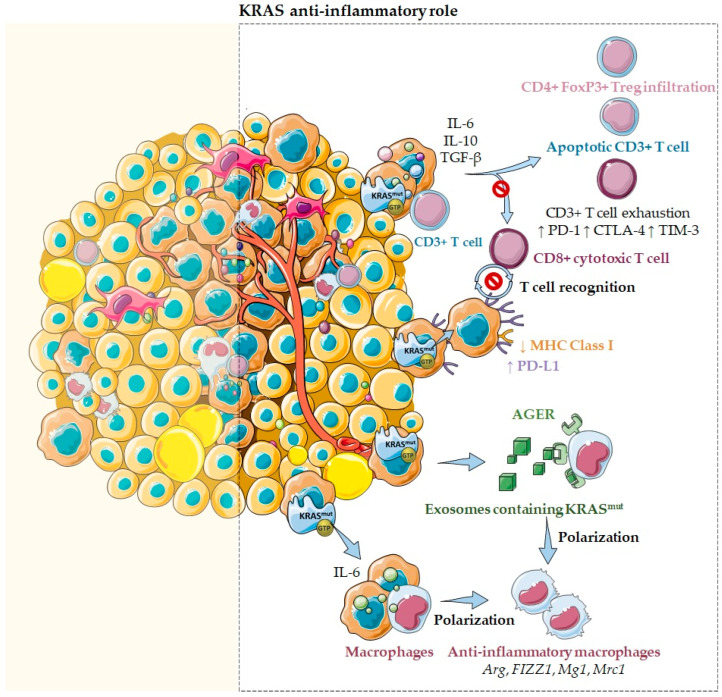
KRAS as an anti-inflammatory tumor microenvironment modulator. Several reports emphasize that *KRAS* mutations may sustain an anti-inflammatory microenvironment through the secretion of several inflammatory chemokines and cytokines, such as TGF-β, IL-10, and IL-6. In fact, cells harboring *KRAS*^G12D^ mutations secrete high levels of these anti-inflammatory mediators that inhibit T cell activation, suppress cytotoxic CD8^+^ T cell-mediated tumor killing, and convert pro-inflammatory CD4^+^ T cells to anti-inflammatory Tregs. Moreover, *KRAS* mutations were also described to induce the downregulation of MHC class I molecules and the upregulation of PD-L1, reducing the ability of CD8^+^ cytotoxic T cells to recognize and kill cancer cells. Additionally, *KRAS* mutations may drive an anti-inflammatory and pro-tumor immune suppressive microenvironment mediated through IL-6 secretion. Notably, when IL-6 was blocked, a reduction of anti-inflammatory macrophage gene expression, such as *Arg*, *FIZZ1*, *Mg1*, and *Mrc1*, and a reduction of the immunosuppressive cytokines TGF-β and IL-10 were observed. Moreover, it has also been described that IL-6 induces higher levels of T cell exhaustion markers, such as PD-1, CTLA-4, and TIM-3. Furthermore, *KRAS* mutations effects can also be mediated through exosomes containing *KRAS*^G12D^. These exosomes can be taken via an AGER-dependent mechanism—a multiligand receptor—by macrophages, modulating their differentiation into a pro-tumor/anti-inflammatory phenotype.

*KRAS* mutations have generally been more related to an anti-inflammatory and, consequently, pro-tumor microenvironment rather than a pro-inflammatory one. However, several studies also reported the association of KRAS with pro-inflammatory/anti-tumor cytokines and chemokines. In fact, *KRAS* mutations have been related to pro-inflammatory chemokines, such as ICAM-1, IL-18, and IL-6. Nevertheless, while the first two have been frequently associated with pro-inflammatory functions [1,32] (Figure 3), IL-6 has been described to exert an anti-inflammatory role in the *KRAS* mutation context (Figure 4) [33].

In the pancreas, normal acinar cells transfected with oncogenic mutant *KRAS* are described to express high levels of ICAM-1, which is then converted to a soluble form, the sICAM-1 [18]. In its turn, sICAM-1 acts as a chemoattractant for immune cells, namely, M1-like/pro-inflammatory macrophages, but not for M2-like/anti-inflammatory ones. Attracted pro-inflammatory macrophages directly interact with acinar cells via membrane ICAM-1, providing enzymes that allow ECM degradation, such as matrix metalloproteinase 9 (MMP-9), as well as inflammatory cytokines and chemokines that can drive transdifferentiation signaling, such as TNF-α (Figure 3). This process is believed to contribute to acinar cells metaplasia and to drive the initiation of precancer lesions, which ultimately can progress to pancreatic cancer [1,18,45]. Overall, these data support KRAS, ICAM-1, and inflammation as contributors to pancreatic ductal adenocarcinoma by initiation and acceleration of acinar-ductal metaplasia [45].

*KRAS* mutations can also modulate the TME through IL-18, which is an immune-stimulatory cytokine [46]. It is an important chemokine produced by epithelial cells of the gastrointestinal tract, the airway, and the skin and also by activated macrophages, Kupffer cells, B cells, and dendritic cells [46]. IL-18 has been implicated in host immune defense against tumor development [46]. Smakman and co-workers demonstrated, using the colorectal cancer cell line C26, that *KRAS* knockdown resulted in the upregulation of IL-18 and its secretion into the medium [1,46]. Authors also evidenced that C26 tumor growth in the liver can be strongly inhibited by the production of IL-18 by hepatocytes [46]. Thus, this work demonstrated that *KRAS*^G12D^ mutation suppresses IL-18 chemokine production, possibly contributing to evasion of the local immune system during tumor development (Figure 3) [1,46].

In lung cancer, to the best of our knowledge, there are no reports concerning the pro-inflammatory functions mediated by KRAS.

### 2.2. KRAS as an Anti-Inflammatory Modulator of the Tumor Microenvironment

Paradoxically, tumors harboring *KRAS* mutations have also been associated with an immunosuppressive and anti-inflammatory microenvironment (Figure 4). In colon cancer, *KRAS*^G12V^ mutants are described to catalyze the differentiation of pro-inflammatory T cells into immunosuppressive T regulatory cells (Tregs) and promote their infiltration in a *KRAS*-driven lung tumorigenesis mouse model [41]. In lung cancer, *KRAS* mutations are associated with high levels of Treg infiltration [41], especially the *KRAS*^G12D^ mutation, which induces CD3^+^ T cell apoptosis and impairs the cytotoxic CD8^+^ T cell activation [41]. Additionally, in pancreatic cancer, cells harboring *KRAS*^G12D^ mutations secrete high levels of the anti-inflammatory mediators TGF-β and IL10, crucial chemokines for sustaining an immunosuppressive environment and cancer cell immune escape [41]. Among their multitude of functions, IL-10 is well-known to inhibit T cell activation, whereas TGF-β inhibits T cell activation and proliferation and promotes epithelial to mesenchymal transition, favoring cancer cell migration and invasion. Additionally, IL-10 and TGF-β released by pancreatic cancer cells are described to suppress cytotoxic CD8^+^ T cell-mediated tumor killing [41]. Moreover, in pancreatic cancer, it was also reported that *KRAS* mutations effects could be mediated through exosomes. In fact, Dai and collaborators reported that exosomes containing *KRAS*^G12D^ are released by dead, dying, or stressed cells, such as cancer cells [44]. These exosomes can be taken via an AGER (advanced glycosylation end product-specific receptor) dependent mechanism—a multiligand receptor—by macrophages. This process causes their differentiation into an M2-like pro-tumor/anti-inflammatory phenotype through the signal transducer and activator of transcription 3 (STAT3)-dependent fatty acid oxidation mechanism (Figure 4) [44].

Other reports emphasize that *KRAS* mutations may sustain an anti-inflammatory microenvironment through the secretion of several inflammatory chemokines and cytokines, such as IL-6, IL-10, and GM-CSF [33].

In fact, high IL-6 secretion was observed in different cell types harboring oncogenic *KRAS* mutations, such as human lung and kidney cells, fibroblasts, and myoblasts [1]. In lung cancer cells, *KRAS* mutations seem to promote increased levels of IL-6 via NF-kB, resulting in the activation of the STAT3 pathway [33]. In its turn, IL-6-mediated STAT3 activation may contribute to immunosuppressive MDSCs accumulation [33]. Paradoxically, IL-6 was also associated with pro-tumor Treg/Th17 cell response due to the observation that anti-IL-6 treatment promotes a T cell response switch, from a pro-tumor Treg/Th17 to an anti-tumor cytotoxic CD8^+^ T cell response [33]. Therefore, IL-6 may re-educate the lung microenvironment towards an anti-inflammatory phenotype, limiting inflammation via polarization of anti-inflammatory macrophages, recruitment of MDSCs and Treg/Th17 increasing response, favoring tumor immune escape and growth [33]. In pancreatic cancer, *KRAS* mutations are present in the majority of the cases, as are high levels of IL-6. In fact, Ras-driven pancreatic tumors are described to promote IL-6 secretion leading to STAT3 signaling pathway activation [5,32]. Interestingly, both molecules are required as mediators of *KRAS* mutations to promote pancreatic cancer precursor lesions initiation and progression to pancreatic ductal adenocarcinoma [1]. Notably, when IL-6 is blocked, a reduction of anti-inflammatory macrophage gene expression, such as Arginase1 (*Arg*), Found in inflammatory zone 1 (*FIZZ1*), Macrophage galactose binding lectin (*Mg1*), and Mannose receptor C type 1 (*Mrc1*), and a reduction of the immunosuppressive cytokines TGF-β and IL-10 were observed. Moreover, a downregulation of the surface expression of the Natural killer group 2 member D receptor (NKG2D or CD314) was also described on NK cells as a mechanism to escape NK cell-mediated cytotoxicity in *KRAS*-driven lung murine models [35]. Additionally, although activated and effector-memory CD8^+^ T cells are described to increase in *KRAS* mutated mouse models, this seems not to be sufficient to impair tumor growth, suggesting the presence of parallel immune escape mechanisms [35]. It has also been described that IL-6 stimulates the recruitment of neutrophils, decreases T-cell infiltration, and induces higher levels of T-cell exhaustion markers, such as programmed cell death 1 (PD-1), cytotoxic T-lymphocyte-associated antigen 4 (CTLA-4), and T-cell immunoglobulin and mucin-domain containing-3 (TIM-3). Altogether, these data demonstrate that *KRAS* mutations may drive an anti-inflammatory and pro-tumor immune suppressive microenvironment mediated through IL-6 secretion (Figure 4).

Nevertheless, IL-10 is also modulated by *KRAS* mutations. In fact, in colorectal cancer cells harboring *KRAS* mutations, an upregulation of the anti-inflammatory cytokine IL-10 via the MEK/ERK/AP-1 pathway was observed. Secreted IL-10 enabled the conversion of pro-inflammatory CD4^+^ T cells to anti-inflammatory CD4^+^ FoxP3^+^ Tregs cells [1,32,47]. In addition, it was described that *KRAS*^G12D^ could promote Treg transformation by blocking the interferon regulatory factor 2 (IRF2), resulting in repression of IRF2/CXCL3 pathway and binding of CXCL3 to CXCR2 on MDSCs, driving immune suppression and immune therapy resistance in colorectal cancer [1,47]. Similarly, in pancreatic cancer, it was also confirmed that IL-10 stimulates the conversion of CD4^+^ T cells to CD4^+^ FoxP3^+^ Tregs cells [1,32,47]. In lung cancer, IL-10 was also reported to mediate the recruitment of anti-inflammatory M2 macrophages and Tregs to the tumor [32].

Interestingly, GM-CSF was also identified as a transcriptional target of oncogenic *KRAS* in pancreatic ductal epithelial cells and in colorectal cancer [1,48,49]. GM-CSF serves as a proliferation and maturation factor of several myeloid cells and has the potential to promote both anti- and pro-inflammatory effects [16]. In pancreatic cancer, GM-CSF is produced in response to activation of KRAS through the concerted action of multiple effectors, such as ERK and PI3K [16]. Additionally, it is related to the expansion of immunosuppressive Gr1^+^ CD11b^+^ myeloid cells. However, GM-CSF is not unique in this ability; IL1-β, IL-6, and VEGF also have this capacity and, curiously, are also targets of oncogenic Ras signaling [16].

Importantly, *KRAS* mutations were also described to induce the downregulation of major histocompatibility complex (MHC) class I molecules, reducing the ability of CD8^+^ cytotoxic T cells to recognize and kill cancer cells (Figure 4) [1].

In addition, *KRAS* mutation status has correlated positively with the programmed death-ligand 1 (PD-L1) expression in distinct cancers [1,36]. In *KRAS* mutant lung cancer cells, oncogenic *KRAS* was proven to upregulate PD-L1 through an increase in PD-L1 mRNA stability mediated by the AU-rich element-binding protein tristetraprolin (TTP) [1]. This expression is regulated by MAPK-dependent transcriptional activity of the activator protein 1 (AP-1) and by STAT3 [1]. More recently, a correlation between *KRAS* mutations, increased PD-L1 expression, and increased CD8^+^ tumor-infiltrating lymphocytes was observed, linking *KRAS* mutations as a promoter of an anti-inflammatory, immunosuppressive TME, adaptive immune resistance, and tumor immunogenicity (Figure 4) [1,36]. Other relevant studies demonstrated that the co-mutation of *TP53* and *KRAS* led to an immune-rich microenvironment of high tumor mutation burden (TMB), enhanced PD-L1 expression, and enrichment of immune cell infiltration, namely, CD4 memory T cells, NK cells, and M1 macrophages. In lung adenocarcinoma, the *TP53*/*KRAS* co-mutation induced an increased expression of PD-L1 [50]. These co-mutations were also reported to play a role in the activation of immune escape and anti-tumor immunity [24,51]. In fact, it was reported that KRAS and TP53 cooperate to promote tumor and immune invasion by activating the ARF6/AMAp1 pathway, which provokes PD-L1 recycling and its cell surface expression [1]. The induction of an immunosuppressive microenvironment orchestrated by *KRAS* mutants seems to be also dependent on the transcription regulator Yap, due to the observation that Yap ablation in *KRAS*/*TP53* mutant pancreatic cells prevents MDSC recruitment favoring MHCII^+^ anti-tumor macrophages, resulting in T cell reactivation, apoptosis of neoplastic cells, and tissue regeneration [51]. In detail, Yap binds to the promoter region of CSFS and of IL-6, controlling their transcription in *KRAS*/*TP53* mutant pancreatic cells. In the absence of Yap, IL-6 and CSFS are blocked, and INF-γ, IL-12, IL-15, IL-4, and IL-13 are produced, stimulating T cell activity [51]. Finally, *KRAS* and *MYC* also cooperate to establish an immune-suppressive stroma through the involvement of CCL9 mediated recruitment of macrophages, PD-L1 and IL-23 dependent exclusion of T and B cells and natural killer (NK) cells [52]. Overall, *KRAS* mutations have a more relevant impact on promoting an anti-inflammatory microenvironment beneficial for tumorigenesis and immune escape than the opposite. The impact of such effects on tumor immune escape and progression is evident, but their contribution to the success of therapeutic response should be further exploited in the near future.

## 3. Therapies Targeting *KRAS* Mutations and the Tumor Microenvironment

**Figure 5:**
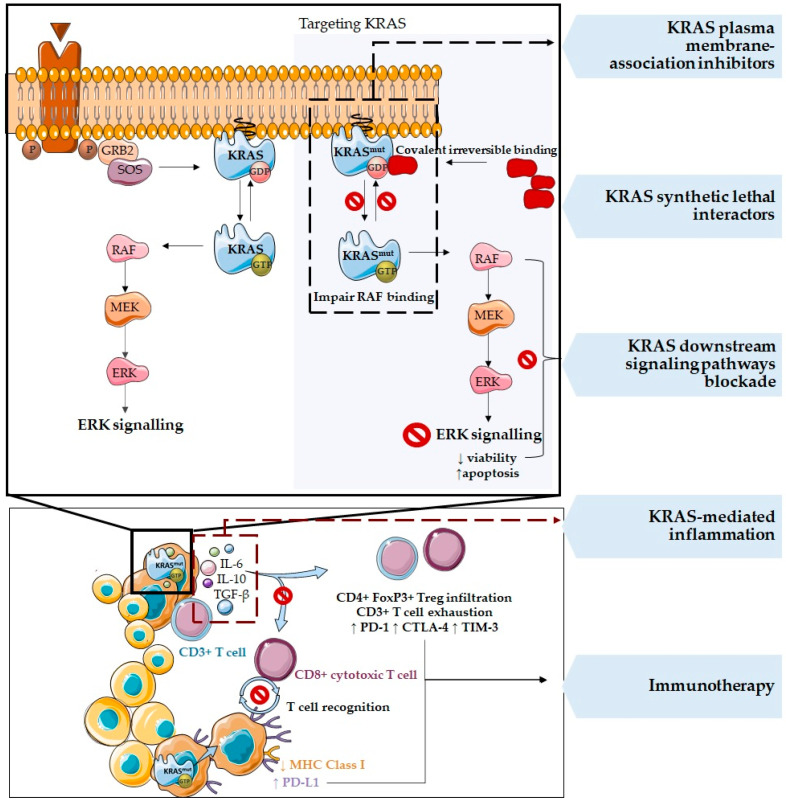
Strategies to target *KRAS* mutations. Several therapies have been developed to target KRAS, namely, KRAS plasma membrane association inhibitors, KRAS synthetic lethal interactors, KRAS downstream signaling pathways blockade, KRAS-mediated inflammation, and immunotherapy. One of the most promising strategies is the novel KRAS synthetic lethal interactors that specifically target the cysteine in the mutated *KRAS*^G12C^ through covalent irreversible binding and favor KRAS-GDP state over GTP. These alterations impair RAF binding and the activation of the signaling pathway, decreasing cell viability and increasing apoptosis of those cells harboring *KRAS*^G12C^ mutations.

Oncogene *KRAS* mutations are a predictive factor for the ineffectiveness of target therapies against epidermal growth factor receptor (EGFR). Despite EGFR antibodies specificity, *KRAS* mutations act downstream EGFR and activate the intracellular RAS pathway, independently of the stimulation via EGFR [53,54,55,56,57]. To overcome this resistance, several therapies have been developed in order to target KRAS, namely, (i) KRAS post-translational modifications; (ii) KRAS synthetic lethal interactors; (iii) KRAS plasma membrane association inhibitors; (iv) downstream signaling pathways blockades, such as RAF and MEK inhibitors; (v) KRAS-regulated metabolic targets, as autophagy and micropinocytosis inhibitors; (vi) KRAS-induced inflammation, such as IL-6 inhibitors; and (vii) immunotherapy (Figure 5) [1,10,36,38,58,59,60,61].

One of the most promising strategies is the novel *KRAS* mutation inhibitors that, specifically target the cysteine residue of mutated *KRAS*^G12C^ through covalent irreversible binding, favoring KRAS-GDP state over GTP. These alterations impair RAF binding and the activation of the signaling pathway, decreasing cell viability and increasing apoptosis of those cells harboring *KRAS*^G12C^ mutations [10,57,62,63]. ARS-853, ARS-1620, MRTX1257, AMG-510 (Sotorasib), and MRTX849 (Adagrasib) are *KRAS*^G12C^ potent inhibitors, AMG-510 and MRTX849 being the first ones to enter in the clinic [57,62,64]. Although *KRAS*^G12C^ is the most frequent mutation in lung cancer with a frequency of 13%, it is present at low percentages in colorectal cancer (3%) and other solid tumors (2%) [63]. Thus, it is imperative to try to target other *KRAS* mutations. Interestingly, AMG-510 and MRTX1257 were reported to induce a pro-inflammatory microenvironment also, suggesting a synergistic effect between this class of inhibitors and the immune checkpoint inhibitors [9,10]. In fact, both AMG-510 and MRTX1257 provoke a TME remodeling, increasing the density of macrophages, dendritic cells, and T cells (Figure 5) [63,64]. Additionally, those drugs drive a change of macrophages phenotype and, most dramatically, CD8^+^ T cell infiltration, favoring a pro-inflammatory/anti-tumor immune response [63,64]. Thus, both treatments prompted a pro-inflammatory microenvironment that could be highly responsive to immune checkpoint inhibitors. Accordingly, recent studies combining AMG-510 with anti-PD-1 immune checkpoint blockade improved survival in a syngeneic *KRAS*^G12C^ mutant CT26 colon carcinoma subcutaneous model [63]. Altogether, these studies demonstrated the relevance of highlighting *KRAS* mutations’ effects on the TME.

## 4. Immunotherapy and Combined Therapeutic Approaches in *KRAS* Mutated Cancers

Immunotherapy targeting immune checkpoint molecules, such as PD-1, PD-L1, and CTLA-4, has also been demonstrated to be one of the most hopeful cancer treatments, with positive results in *KRAS* mutated cancers, as further described in [38,60]. PD-1, which binds to PD-L1, also known as CD274, or to PD-L2/CD273, is a peripheral immune checkpoint of immune, tumor, and stromal cells, whereas CTLA-4 binds to CD80/CD86, also known as B7-1/B7-2 co-stimulatory receptors, on antigen-presenting cells (APCs) [65].

**Figure 6:**
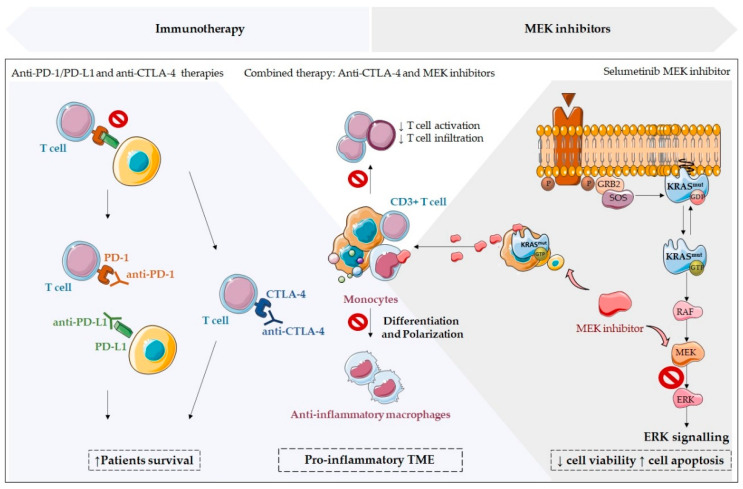
Combined therapeutic approach in *KRAS* mutated cancers: immunotherapy and MEK inhibitors. Immunotherapy targeting immune checkpoint molecules, such as PD-1, PD-L1, and CTLA-4, has been demonstrated to be one of the most hopeful cancer treatments, with positive results in *KRAS* mutated cancers. Surprisingly, the combined therapies of MEK inhibitors with antibodies targeting PD-1, PD-L1, or CTLA-4 exert higher anti-tumor effects than monotherapies. In a murine *KRAS*-mutant colorectal cancer model, the MEK inhibitor selumetinib attenuated anti-CTLA-4-mediated T cell activation and infiltration into tumors and blocked monocytes differentiation into anti-inflammatory macrophages. Thus, MEK inhibition, specifically selumetinib, brings beneficial effects to the TME in the context of CTLA-4 blockade, and, more importantly, this combination of MEK inhibitors with CTLA-4 blocking antibodies re-educates the TME from an immunosuppressive to an immune alert status, expanding therapeutic intervention.

Specifically, in advanced-stage non-small-cell lung carcinoma patients, monoclonal antibodies that target PD-1 and its main ligand PD-L1 have shown survival increments demonstrating the favorable clinical benefits of anti-PD-1/PD-L1 immunotherapy (Figure 6) [36,66]. Thus, anti-PD-1/PD-L1 therapies in lung cancer were already approved [60]. However, anti-CTLA-4 antibodies did not present encouraging results in lung carcinoma [60]. In colorectal cancer, anti-PD-1/PD-L1 therapy, such as Pembrolizumab, was also approved in a subgroup of patients, namely those harboring mismatch repair (MMP) deficient tumors, which present higher PD-L1 expression, in comparison to MMP proficient carcinomas [57,60,65,67,68]. In addition, Nivolumab, another PD-1 blocking antibody, was also FDA approved for MMR and MSI-H metastatic CRC treatment [65]. In pancreatic cancer, these immunotherapies demonstrated limited clinical success and, for this reason, immunotherapy is not included in the clinical guidelines in this type of cancer [60,61]. Moreover, it was already demonstrated that not all *KRAS* mutations can benefit from immunotherapy, as there are differences in immunotherapy efficacy among *KRAS* mutant subtypes, namely, *KRAS*^G12D^ mutations [69]. Overall, these results seem disappointing. However, *KRAS* mutations and their complex impact on the TME may explain the ineffectiveness of these immunotherapy treatments. For this reason, exploring *KRAS* mutations impact on TME can bring a new vision of combined therapeutic approaches to overcome KRAS-associated therapeutic inefficiency and/or resistance. In fact, *KRAS* mutations and other interactors have been already explored as possible genetic markers to distinguish patients who may benefit from immune checkpoint inhibitors, such as (i) *TP53* co-mutation; (ii) functional mismatch repair; (iii) PD-L1 expression; (iv) the intensity of CD8^+^ T cell infiltration [4,50,67,69,70]. In detail, *TP53/KRAS* co-mutation could increase the tumor mutation burden of all *KRAS* mutants, except the *KRAS*^G12D^ mutation subtype, transforming *TP53/KRAS* mutated cancers more responsive to immunotherapy [69]. Specifically, in colorectal cancer, the different impact of the *KRAS*^G12D^ mutation subtype was explored, and IRF2-CXCL3 pathway blocking was identified as a driver of immune suppression and immune therapy resistance [69]. Additionally, IRF2 overexpression was also explored, and it was verified that this strategy overcomes KRAS-induced therapy resistance to anti-PD-1 immunotherapy, pointing to this molecule as a potential therapeutic co-target in KRAS-induced cancers [1]. Overall, these studies are a clear example of the urgency to understand why some patients still do not respond to immunotherapy, or other therapies, to develop novel strategies to overcome resistance. 

Encouragingly, combined strategies have also been proposed to overcome acquired resistance and/or ineffectiveness, namely, targeting oncogenic signaling pathways and the microenvironment [1,36,60]. Despite the lack of clinical relevance in some cases, the combined therapies of MEK inhibitors, with antibodies targeting PD-1, PD-L1, or CTLA-4, have been explored and demonstrated to exert higher anti-tumor effects than monotherapies [1,60,71]. In fact, in a murine *KRAS*-mutant colorectal cancer model, the MEK inhibitor selumetinib seemed to attenuate anti-CTLA-4-mediated T-cell-activation and infiltration into tumors without abrogating these effects. Specifically, selumetinib reduced CD11b^+^ Ly6G^+^ tumor-infiltrating neutrophils or Granulocytic-Myeloid-derived suppressor cells (gMDSC), and blocked monocytes differentiation into anti-inflammatory macrophages. MEK inhibitors also seemed to reverse the anti-CTLA-4-mediated induction of two key immunosuppressive factors, Arg1 and cyclo-oxygenase-2 (Cox-2) [71]. Thus, MEK inhibition, specifically selumetinib, brings beneficial effects to the TME in the context of CTLA-4 blockade [71]. These authors also demonstrated that selumetinib led to CD11b^+^ Ly6C^+^ MHCII^+^ cells accumulation, a subset of myeloid cells related to an intermediate state of infiltrating monocytes differentiation into macrophages. Thus, MEK inhibition prevented macrophage accumulation through impairment of monocyte differentiation to macrophage [71]. In addition, the combination of MEK inhibitors with CTLA-4 blocking antibodies re-educates the TME from an immunosuppressive to an immune alert status, expanding therapeutic intervention (Figure 6) [71].

## 5. Final Conclusions

In summary, there is a high prevalence of oncogenic *KRAS* mutations in cancers, being more evident in pancreatic, colorectal, and lung cancers. Interestingly, *KRAS* mutations have been tightly associated with modulation of inflammation, which has been gradually recognized as a key contributor for tumorigenesis by affecting immune response, as well as the efficacy of treatments [1,40]. Here we summarize the intensive efforts that have been made to understand *KRAS* mutations effects, not only on cancer cells, but also on the TME. Additionally, with the exploration of *KRAS* mutations impact on the inflammatory TME, we highlight new avenues for investigating the potential of these mutations on the TME modulation, opening a new vision of combined therapeutic approaches to overcome KRAS-associated therapy inefficacy and/or resistance.

## Data Availability

Not applicable.
